# Cytogenomic results following high-chance non-invasive prenatal testing: a UK national audit

**DOI:** 10.1017/S0016672320000087

**Published:** 2020-09-01

**Authors:** Fiona S. Togneri, Stephanie K. Allen, Kathy Mann, Elaine Holgado, Sian Morgan

**Affiliations:** 1 West Midlands Regional Genetics Laboratory, Birmingham Women's and Children's NHS Foundation Trust, Edgbaston, B15 2TG, UK; 2 Genetics Department, Viapath Analytics, Guy's Hospital, London, SE1 9RT, UK; 3 Molecular Genetics, TDL Genetics and Health Services Laboratories, London, UK; 4 All Wales Genetics Laboratory, Institute of Medical Genetics, University Hospital of Wales, Cardiff, Wales, UK

**Keywords:** amniocentesis, pregnancy, prenatal diagnosis, trisomy, ultrasound

## Abstract

**Objective:**

Non-invasive prenatal testing (NIPT) is increasingly being adopted as a screening test in the UK and is currently accessed through certain National Health Service healthcare systems or by private provision. This audit aims to describe reasons for and results of cytogenomic investigations carried out within UK genetic laboratories following an NIPT result indicating increased chance of cytogenomic abnormality (‘high-chance NIPT result’).

**Method:**

A questionnaire was sent out to 24 genetics laboratories in the UK and completed by 18/24 (75%).

**Results:**

Data were returned representing 1831 singleton pregnancies. A total of 1329 (73%) invasive samples were taken following NIPT results showing a high chance of trisomy 21; this was confirmed in 1305 (98%) of these by invasive sampling. Trisomy 21 was confirmed in >99% of patients who also had high-screen risk results or abnormal scan findings. Amongst invasive samples taken due to NIPT results indicating a high chance of trisomy 18, 84% yielded a compatible result, and this number dropped to 49% for trisomy 13 and 51% for sex chromosomes.

**Conclusion:**

In the UK, the majority of patients having invasive sampling for high-chance NIPT results are doing so following an NIPT result indicating an increased chance of common trisomies (92%). In this population, NIPT performs particularly well for trisomy 21, but less well for other indications.

## 1. Introduction

Since its introduction, non-invasive prenatal testing (NIPT) has been enthusiastically adopted as a prenatal screen across the globe (Minear *et al.*, 2015). The UK National Screening Committee in 2016 recommended the evaluative implementation of NIPT within the National Health Service (NHS) (UK National Screening Committee, 2016); however, at present, NIPT is available as a nationally funded contingent screen in Wales only (Health Wales, 2018). Access to NIPT across the rest of the UK is currently inconsistent. Some NHS Trusts are offering local services (Wald *et al.*, 2018; Togneri *et al.*, 2019; Sacco *et al.*, 2020), but much NIPT is being obtained on a private basis. All NIPT providers test for trisomy 13, 18 and 21; however, only a proportion test for sex chromosome aneuploidy, other autosomal aneuploidies and structural chromosome abnormalities such as microdeletion syndromes. Despite much NIPT being obtained on a private basis, patients who receive a high-chance result usually access genetic counselling and follow-up invasive procedures through NHS healthcare services. Invasive samples (amniocentesis or chorionic villus samples) taken for genetic diagnosis are sent to 1 of 24 mainly NHS-based UK genetics laboratories for analysis.

The main objectives of this study were to determine the number of invasive procedures received through the UK healthcare system prompted by NIPT, to evaluate the reasons for referral and to determine rates of confirmation of the suspected diagnosis.

## 2. Material and methods

The Association of Clinical Genomic Science (ACGS) conducted a UK national survey at the end of 2018 concerning invasive prenatal samples received by UK genetics laboratories following NIPT results that show a high chance of genomic imbalance. The data covered all invasive samples received by genetics laboratories since the introduction of NIPT in the UK. The survey was drawn up by three members of the ACGS with involvement in prenatal diagnosis in the NHS and current NHS NIPT services in the UK and approved by the chairs of the ACGS scientific and technology committee. The survey asked for data pertaining to individual test reports, the year in which the sample was received, the type of invasive sample received, the result of genetic diagnosis, the indication for NIPT (where known), the NIPT results (where known), the ultrasound scan findings if detailed, the year of maternal birth and a laboratory sample identifier. A comments box was also provided for any other pertinent details that laboratories wished to provide. After the initial survey was sent out, a single follow-up was sent out after a gap of 2 months. Data were collated and anonymized with a code distinguishing centre of origin assigned to each sample prior to analysis.

## 3. Results

Of the 24 centres contacted, 18 completed and returned the survey (75%). Data were provided for a total of 1831 invasive samples from singleton pregnancies (1083 amniotic fluid samples and 748 chorionic villus samples), all taken following NIPT results indicating a high chance of genomic imbalance or following failed NIPT studies. The median number of samples received per centre was 81 (range: 16–509). Laboratories first received invasive samples following a high-chance NIPT result in 2013. Nine invasive samples were received nationally in 2013, and this rose to 519 in 2018 (Table 1). A median maternal age of 35 years was observed (range: 21–55 years).

**Table 1.** Full data table showing the reasons for invasive prenatal samples being taken following non-invasive prenatal testing (NIPT) and rates of discordancy (singleton pregnancies only).

Metric	Patient population, *n* (%)	Discordant results following invasive sampling, *n* (%)
*Total number of invasive samples*	*1831*	*193* (*10.5)*
Amniotic fluid	1083 (59)	143 (13)
Chorionic villus	748 (41)	50 (7)
*Year*		
2013	9	
2014	210	
2015	272	
2016	376	
2017	445	
2018	519	
NIPT result		
*Common autosomal aneuploidy*	*1682 (92)*	*120* (*7)*
Trisomy 21	1329	24 (2)
Trisomy 18	242	39 (16)
Trisomy 13	111	57 (51)
*Sex chromosome aneuploidy*	*114 (6)*	*56* (*49)*
Turner syndrome	61	34 (56)
Triple X	21	10 (48)
Klinefelter syndrome	28	12 (43)
XYY	3	0
XXYY	1	0
*Other genomic imbalance*	*21 (~1)*	*17* (*81)*
22q11.2 microdeletion	7	6 (86)
Other CNV	5	2 (40)
Rare autosomal aneuploidy (all AFs)	7	7
Triploidy (both CVS)	2	2
*Fail*	**14 (~1)**	
Trisomy 21 (one with scan findings)	*2*	
No aneuploidy detected	*10*	
Other genomic imbalance	*2*	
Samples with stipulated clinical indication for NIPT	**777 (42)**	**31** (**4)**
*High risk from ANS*	*404 (52)*	*21* (*5)*
Trisomy 21	339	2 (0.6)
Trisomy 18	41	6 (15)
Trisomy 13	14	10 (71)
Turner syndrome	5	3 (60)
*Previous trisomy*	*10 (1)*	*2* (*20)*
*Ultrasound scan findings*	*363 (47)*	*6* (*1.7)*
Trisomy 21	261	2 (0.8)
Trisomy 18	70	1 (1.4)
Trisomy 13	22	1 (4.5)
Turner syndrome	8	2 (25)

AF = amniotic fluid; ANS = antenatal screening; CNV = copy number variation; CVS = chorionic villus sampling.

A clinical indication for NIPT was stipulated for 777 (42%) patients, with a median maternal age of 37 years observed for these samples. Of these patients, 52% had received a high-screen-risk result from current NHS biochemical screening pathways (median maternal age of 38 years); a further 47% of pregnancies had abnormal ultrasound scan findings with patients initially declining invasive sampling (median maternal age of 40 years). The remaining 1% of patients had experienced previous trisomic pregnancies (median maternal age of 44 years) (Table 1; maternal age data not shown).

Twelve samples were referred as a result of NIPT results indicating double trisomies. Double trisomy was confirmed in one patient, seven showed single aneuploidies only and in four patients no aneuploidy was confirmed.

A total of 92% (1682) of invasive samples were taken following NIPT results showing a high chance of common autosomal trisomies (Table 1). The remaining patients had less common indications from NIPT. These included sex chromosome aneuploidies (6%), rare autosomal aneuploidies, other genomic imbalances (such as microdeletion syndromes) and failed NIPT studies (Table 1).

Overall, 76% of patients accessing genetic diagnoses were doing so due to NIPT results showing a high-chance result for trisomy 21; 98% of these pregnancies were confirmed as having foetal trisomy 21. This number rose to a give a positive predictive value (PVV) of 99% in the high-screen-risk population (patients accessing NIPT following high-chance results from NHS-provided biochemical screening pathways; contingent screen; Table 1) and in patients with abnormal scan findings.

A total of 16% of samples taken due to NIPT results indicating a high chance of trisomy 18 yielded normal cytogenomic results following invasive sampling, and this figure was 51% for results indicating a high chance of trisomy 13 (70% of the discordant trisomy 13 invasive samples were amniocenteses).

Approximately 50% of samples taken following NIPT results for a high chance of sex chromosome aneuploidy also yielded euploid cytogenomic results (79% of these were amniocenteses).

In total, 363 patients having invasive sampling following NIPT results also had abnormal ultrasound scan findings. NIPT results were confirmed in >98% of these patients following cytogenomic procedures (PPV of 99.2% for trisomy 21, 98.6% for trisomy 18 and 95.5% for trisomy 13).

A total of 21 invasive prenatal samples were taken across the past 6 years as a result of less common NIPT high-chance findings, such as rare autosomal aneuploidies, triploidy and copy number variations, including 22q11.2 microdeletion syndrome; 17 (81%) yielded normal euploid results.

A further 14 invasive samples were taken as a result of failed NIPT studies. Of those, four were shown to have genomic imbalance. For the majority of these patients, the reason for NIPT was not stipulated; three patients had an increased risk from biochemical screening and one patient had an abnormal ultrasound scan finding. The reason for NIPT failure was also not given.

Data were also provided for 19 twin pregnancies (Table 2). In four of these pregnancies, trisomy was confirmed in both twins (2 × trisomy 18 and 2 × trisomy 21). In the remaining 15 pregnancies, trisomy was confirmed in one twin only (1 × trisomy 18 and 14 × trisomy 21). Reason for NIPT was stipulated for nine of these pregnancies; three pregnancies had abnormal scan findings and six had an increased screen risk from combined first-trimester screening.

**Table 2.** Data pertaining to invasive samples received for twin pregnancies following high-chance non-invasive prenatal screening (NIPT) results.

Metric	Patient population, *n* (%)	Discordant results following invasive sampling
*Total number of twin pregnancies*	*19*	*0^a^*
Amniotic fluid	10 (53)	
Chorionic villus	9 (47)	
Reason for NIPT		
Not stipulated	10	
Screening risk	6	
Ultrasound scan findings	3	
NIPT result		
Trisomy 18	3	
Trisomy 21	16	
Twin concordance		
Both twins trisomic	4	
One twin trisomic	15	

*^a^*Aneuploidy confirmed in at least one twin; for most pregnancies, invasive samples were also received for the other twin.

## 4. Discussion

This is the first UK-wide study describing the population of patients requesting invasive prenatal genetic diagnosis within the UK healthcare system following NIPT results that indicate a high chance of genomic imbalance or an inconclusive result. A 75% response rate for the survey reflects the engaged genetics community in the UK. Non-responding laboratories are likely to have received very few samples fitting the criteria for this survey. In summary, of the 1831 samples (chorionic villus sampling and amniocentesis) received by UK genetics laboratories from singleton pregnancies following a high-chance NIPT result, 1638 (89.5%) were confirmed to have genomic imbalance. This illustrates the fact that NIPT is a screening test and that, in clinical practice, amniocentesis or chorionic villus sampling is an important confirmatory test for all high-chance NIPT results.

Audit data show that the majority of patients accessing invasive sampling are doing so due to NIPT results indicating a high chance of common autosomal trisomies or sex chromosome aneuploidies. NIPT performs particularly well when trisomy 21 is identified and better than predicted in the literature (Taylor-Phillips *et al.*, 2016; van der Meij *et al.*, 2019). This is particularly notable given that UK NIPT provision is by both NHS and a number of private providers and therefore more heterogeneous than in other countries. Foetal trisomy 21 was confirmed in most women where the pregnancy had been identified to have both a high-risk biochemical screening result followed by a high-chance NIPT result (PPV of 99.4%).

Trisomy 13 was confirmed in fewer than 50% of invasive samples following high-chance trisomy 13 NIPT results (consistent with data from other populations; van der Meij *et al.*, 2019); this increased to 95.5% in the presence of abnormal foetal scan findings. In total, 40/57 discordant invasive samples taken for confirmation of trisomy 13 findings were amniotic fluid samples, and confined placental mosaicism (Grati *et al.*, 2015) is considered likely to be a major cause of this discordancy, together with other biological and technical explanations. The smaller number of referrals for high-chance sex chromosome aneuploidy or microdeletions in part reflects the limited availability of these NIPT tests in the UK.

While it is not recommended for NIPT to be offered in place of invasive sampling for genetic evaluation of the aetiology of ultrasound anomalies (Beulen *et al.*, 2017), in the UK many clinicians will offer NIPT to patients initially declining invasive sampling. This practice has been shown to be of some benefit to patient care in this patient population (Togneri *et al.*, 2019). Data were received for 363 patients having invasive sampling following high-chance NIPT results and with known ultrasound scan findings suggestive of being associated with a particular trisomy, and NIPT results were confirmed in 98.3%.

In the few foetuses where trisomy was not confirmed by the diagnostic test despite abnormal scan findings being present together with the high-chance NIPT result, it is interesting to note that the ultrasound scan findings in these cases were non-specific (2 × high-chance trisomy 21 results in foetuses with hydrops, 1 × high-chance trisomy 18 result in a foetus with increased nuchal translucency and 1 × high-chance trisomy 13 and 2 × high-chance Turner syndrome results in foetuses with isolated cardiac defects).

Data were provided for 19 twin pregnancies. In all of these pregnancies, trisomy was confirmed in one or both twins following invasive sampling. While these data are very limited, they do support the offering of NIPT for this population where the pregnancy has been identified as high risk.

Invasive sampling following high-chance NIPT results for other less common genomic imbalance is currently rare due to the limited availability of these tests; follow-up invasive testing found a high incidence of false-positive results. NIPT results indicating a high chance of sex chromosome aneuploidies were confirmed in only ~50% of patients. The numbers in this category are relatively low, as NIPT for sex chromosome aneuploidy is not recommended within the UK antenatal screening programme. It could be argued that testing for the majority of these findings is not clinically indicated, although it may provide beneficial information in cases where clinical indications suggest Turner syndrome and the patient chooses to avoid invasive testing. Very few invasive prenatal samples were received following NIPT results that indicate a high chance for microdeletion syndromes, triploidy or rare autosomal aneuploidy: 21 patients nationwide across a 6-year period. Data support the UK National Screening Committee plan for the evaluative implementation of NIPT for common autosomal trisomies only.
